# 非小细胞肺癌免疫检查点抑制剂假性进展的研究进展

**DOI:** 10.3779/j.issn.1009-3419.2024.101.10

**Published:** 2024-04-20

**Authors:** Yajun TONG, Yong LONG, Fan ZHANG, Junfeng LI

**Affiliations:** ^1^414000 岳阳，岳阳市中心医院肿瘤科; ^1^Department of Oncology, Yueyang Central Hospital, Yueyang 414000, China; ^2^415000 常德，湘雅常德医院肿瘤科; ^2^Department of Oncology, Xiangya Changde Hospital, Changde 415000, China

**Keywords:** 肺肿瘤, 免疫检查点抑制剂, 假性进展, 免疫治疗, Lung neoplasms, Immune checkpoint inhibitors, Pseudoprogression, Immunotherapy

## Abstract

免疫检查点抑制剂的出现极大地改善了晚期肺癌患者的预后，但其可能会导致假性进展，使临床评估和管理变得复杂化。假性进展表现为肿瘤暂时性增大或出现新病变等，继续治疗后影像学上出现改善，多不伴有临床症状恶化。目前假性进展在早期诊断方面仍存在困难，其发生机制尚不明确，缺乏良好的预测因素和相关生物标志物。本文通过对非小细胞肺癌免疫检查点抑制剂假性进展的研究现状进行综述，以期为使用该类药物治疗患者的临床医生提供有益的参考。

## Abstract (en)

The advent of immune checkpoint inhibitors (ICIs) has greatly improved the prognosis of advanced lung cancer patients, but can lead to pseudoprogression (PsP), which complicates clinical evaluation and management. PsP is manifested as temporary enlargement of the tumour or the appearance of new lesions, etc., and improvement in imaging occurs with continued treatment, mostly without worsening of clinical symptoms. Currently, there are still difficulties in the early diagnosis of PsP, and its occurrence mechanism is not yet clear, lacking good predictive factors and related biomarkers. This article reviews the current research status of PsP of ICIs in non-small cell lung cancer in order to provide helpful clinical strategies for oncologists using these drugs.

免疫检查点抑制剂（immune checkpoint inhibitors, ICIs）近年来在黑色素瘤、肺癌、泌尿系肿瘤等实体肿瘤中应用越来越广泛。免疫治疗使驱动基因阴性非小细胞肺癌（non-small cell lung cancer, NSCLC）患者的生存预后和生活质量得到了明显的提升^[1⇓-3]^，同时也增加了毒副反应，给临床医师带来一些挑战。与传统的细胞毒性化疗药物不同，这些药物的反应模式可能是非常规的，包括假性进展（pseudoprogression, PsP）现象，临床上应与真性进展（true progressive disease, TPD）相鉴别，两者在后续治疗或预后方面存在较大的差异。本文从定义、评价标准、临床特点、潜在的发生机制和诊断方法等方面对PsP的研究进展进行综述。

## 1 PsP定义和评价标准

ICIs与传统的细胞毒性化疗药物不同，可能造成一些非典型性反应，包括PsP、超进展（hyperprogressive disease, HPD）和分离反应等。2016年更新了实体瘤疗效评估标准（Response Evaluation Criteria in Solid Tumors, RECIST）^[4]^，但内容中缺少对PsP定义和判断标准的描述。为满足客观评价免疫治疗疗效的需要（表1），多个与免疫治疗相关的放射学评估标准相继颁布。虽然PsP目前没有统一的定义，但通常认为是免疫治疗启动后肿瘤最初尺寸增大或出现新病灶，随后肿瘤负荷下降或稳定的现象^[9,10]^。PsP不是真正意义上的疾病进展（progressive disease, PD），大多数情况下不伴随临床症状恶化。

**表 1:** 评估免疫反应的放射学标准

Criteria	CR	PR	SD	PD	Confirmation of PD	New lesions
RECIST 1.1^[4]^ Uni-dimensional ≥10 mm; 5 lesions in total, 2 per organ	Disappearance of all lesions	≥30% decrease from baseline	Neither CR/PR nor PD	≥20% increase in the nadir of the sum of target lesions (with a minimum of 5 mm)	Not applicable	PD
irRC^[5]^ Bi-dimensional 5x5 mm; 15 lesions in total, 5 per organ	Disappearance of all lesions	≥50% decrease from baseline	Neither CR/PR nor PD	≥25% increase in the nadir of the sum of target lesions	At least 4 weeks later	Incorporated in the sum of measurement
irRECIST^[6]^ Uni-dimensional ≥10 mm; 5 lesions in total, 2 per organ	Disappearance of all lesions	≥30% decrease from baseline	Neither CR/PR nor PD	≥20% increase in the nadir of the sum of target lesions (with a minimum of 5 mm)	At least 4 weeks after and up to 12 weeks	Incorporated in the sum of measurement
iRECIST^[7]^ Uni-dimensional ≥10 mm; 5 lesions in total, 2 per organ	Disappearance of all lesions	≥30% decrease from baseline	Neither CR/PR nor PD	≥20% increase in the nadir of the sum of target lesions (with a minimum of 5 mm)	At least 4 weeks after and up to 8 weeks	iUPD; not incorporated in the sum becomes iCPD if confirmed
imRECIST^[8]^ Uni-dimensional ≥10 mm; 5 lesions in total, 2 per organ	Disappearance of all lesions	≥30% decrease from baseline	Neither CR/PR nor PD	≥20% increase in the nadir of the sum of target lesions (with a minimum of 5 mm)	At least 4 weeks later	Incorporated in the sum of measurement

RECIST: Response Evaluation Criteria in Solid Tumors; irRC: immune-related Response Criteria; irRECIST: immune-related Response Evaluation Criteria in Solid Tumors; iRECSIT: immune Response Evaluation Criteria in Solid Tumors; imRECIST: immune-modified Response Evaluation Criteria in Solid Tumors; CR: complete response; PR: partial response; SD: stable disease; PD: progressive disease; iUPD: immune unconfirmed PD; iCPD: immune confirmed PD.

### 1.1 实体瘤免疫疗效评估标准

RECIST 1.1是最常用的实体瘤疗效评价标准，在该标准中，目标病灶最大直径之和增大≥20%或出现新病灶，即判定为PD，不考虑PsP的情况。为了准确识别免疫治疗的非典型反应模式，免疫相关反应标准（immune-related Response Criteria, irRC）^[5]^于2009年首次提出，该标准基于二维测量，通过测量所有目标病灶的最大垂直直径总和（sum of the perpendicular diameters, SPD）评估肿瘤负荷，最多10个目标病灶且每个器官不超过5个，且允许新病灶计入“总肿瘤负荷”。irRC的核心创新之处在于将可测量的新病变纳入“总肿瘤负荷”并将该变量与基线测量值进行比较，并非直接认定为进展。免疫相关PD（immune-related PD, irPD）定义为肿瘤负荷相对于最低点（记录的最低肿瘤负荷）增加≥25%，自首次记录之日起至少4周内通过重复、连续评估确认。该标准建议对首次评估为irPD且临床未出现明显恶化的患者继续使用免疫治疗，再次评估与最低肿瘤负荷相比未达到irPD者，可认为是PsP。由于irRC采用的是二维测量，与既往研究中采用一维测量标准难以进行直接比较，且目标病灶多，评估实施较为麻烦。在此标准基础上，采用一维测量的实体瘤免疫相关疗效评价标准（immune-related Response Evaluation Criteria in Solid Tumors, irRECIST）在2013年发布，重复性也比二维测量的irRC更好^[6]^。2017年发表了实体肿瘤免疫疗效评价标准（immune Response Evaluation Criteria in Solid Tumors, iRECIST）^[8]^，该标准提出了免疫未确认进展（immune unconfirmed progressive disease, iUPD）和免疫确认进展（immune confirmed progressive disease, iCPD）的两种PD模式。iUPD符合RECIST 1.1标准中PD的定义，但在4-8周内的随访影像评估中未得到确认。iCPD定义为在iUPD后4-8周内的随访中出现新病灶或目标病灶测量值总和进一步增长≥5 mm，非靶病灶符合同样的原则。如果iUPD后下一次评估肿瘤的大小或范围没有发生变化，则在该时间点继续评价为iUPD；肿瘤的变化如果符合RECIST 1.1标准可评价为免疫完全缓解（immune complete response, iCR）、免疫部分缓解（immune partial response, iPR）或免疫疾病稳定（immune stable disease, iSD）。该标准将新病灶单独评估，并未纳入目标病灶测量值总和。指南建议，只要iUPD在下次评估时未确认为iCPD，且临床状态稳定或改善，继续治疗至下次评估，在随后的治疗中出现反应的患者可认定为PsP。2018年发布的实体肿瘤免疫修饰疗效评价标准（immune-modified Response Evaluation Criteria in Solid Tumors, imRECIST）^[8]^中，非目标病变的进展并不能定义PD，但其完全消失有助于定义CR，新病灶虽不能直接定义为PD，但可以通过测量新病灶来计算总肿瘤负荷。imRECIST与irRECIST基本相似，新颖之处主要包括更详细地定义了无进展生存期（progression-free survival, PFS），以及对几种癌症预后关系的评估，imRECIST PD或者死亡被认为是imPFS（imRECIST-defined PFS）事件，但需要排除在随后的时间点（4周后）评估为imRECIST SD/PR/CR的情况。imRECIST PD后续没有进行评估，也应该认为是imPFS事件。如果患者没有出现快速进展，并具有良好的体能状态和耐受性，持续的ICIs治疗仍然可以获得临床获益，甚至达到缓解。

### 1.2 不同标准下的PsP定义

由于目前缺乏对PsP的统一定义，各临床研究采用了不同的界定标准。Park等^[11]^检索并分析了17项研究，对3402例患者进行了荟萃分析，在检索到的文献中，发现至少采用了4种不同的PsP定义：（1）3项研究采用irRC标准，首次评价为irPD后，下次放射学评估达到irCR、irPR、irSD；（2）采用RECIST 1.1标准的研究有5项，PsP定义为首次评价为PD后，继续影像学评估，再次评价达到SD、PR或CR；（3）采用RECIST 1.1标准定义PsP的研究有6项，定义为评价为PD后再次评价达到CR或PR；（4）采用RECIST 1.0标准的研究有3项，PsP被定义为评价PD后再次评价达到SD、PR或CR。结果显示第（1）种定义的研究汇总后PsP发生率最高，为8.0%（95%CI: 5.9%-10.0%），而第（4）种定义的研究汇总后PsP发生率最低，为4.5%（95%CI: 2.0%-7.0%）。Mönch等^[12]^回顾性分析接受ICIs治疗不同实体瘤的PsP模式，采用iRECIST标准评估治疗反应，PsP定义为在后续随访未能证实的iUPD。共纳入834例接受了3次以上影像学评估的免疫治疗实体瘤患者，发现32例患者符合PsP诊断标准，发生率为3.8%。

### 1.3 各评价标准的应用价值

传统的RECIST标准无法识别NSCLC免疫治疗PsP和其他非典型反应，而irRC因为采用二维测量，操作繁琐，重复性差，这两者在免疫治疗疗效评价中具有明显的缺陷。irRECIST和imRECIST标准将新发病灶纳入总肿瘤负荷中，相较于iRECIST标准，总肿瘤负荷增加的幅度更为显著，另外在发生分离反应（如目标病灶缩小，新病灶增大）时，因为肿瘤异质性致使继续使用免疫治疗无效，而这两个标准重复测量的总肿瘤负荷无法识别为进展^[11]^，这种情况在iRECIST标准中可准确判定为iCPD。虽然这些新的标准可以识别PsP，但其发生率低（2%-7%）^[13⇓⇓⇓⇓⇓⇓⇓⇓⇓⇓-24]^，而且PsP在这些新标准中是一种回顾性诊断，可能会让真正进展的患者继续使用无效的免疫治疗，这导致新标准的功效并没有真正优于传统的RECIST标准^[13]^。另外，这些新标准主要为临床试验而制定，并未对PsP的定义进行详细描述，也未设定具体评估细则，而在不同研究中，对PsP采用的定义标准也不一样。在临床诊疗中，我们应该谨慎地根据这些标准来停止ICIs治疗。

## 2 PsP临床相关特征的研究进展

肿瘤组织学、吸烟史、年龄和细胞程序性死亡配体1（programmed cell death ligand 1, PD-L1）表达并不能预见PsP的易感性^[13,23⇓-25]^。临床医师通过了解PsP的发生率、发生时间、表现形式等，有助于在免疫治疗期间准确识别PsP，避免漏诊误诊。

### 2.1 PsP发生率

表2列出了NSCLC免疫治疗PsP有关的研究，大部分研究采用RECIST 1.1标准进行疗效评价，并通过计算机断层扫描（computed tomography, CT）/磁共振显影（magnetic resonance imaging, MRI）对PsP进行回顾性诊断。尽管各项研究均为事后分析，而且PsP的评判标准和定义并不一致，总体来说，NSCLC ICIs治疗后发生PsP的概率较低（0.8%-6.7%）。NSCLC免疫治疗导致的PsP主要发生于使用ICIs单药的患者，在联合治疗中鲜有报道。接受ICIs和脑转移灶放疗的患者属于特殊情况，ICIs联合脑转移灶立体定向放射治疗（stereotactic radiosurgery, SRS）后PsP的发生率为20%，明显高于免疫单药治疗^[26]^，这可能与脑实质接受放疗后的特异性改变相关，如血管内皮损伤、脱髓鞘病变增加、脑肿瘤更容易坏死水肿等。ICIs联合化疗后PsP发生率明显低于免疫单药，目前仅查阅到了2例NSCLC化疗联合ICIs治疗出现PsP的报道^[27,28]^。这种低发生率可能是因为细胞毒性药物在治疗初期就有较强的肿瘤杀灭作用，覆盖了ICIs启动时较弱的抗肿瘤免疫效应阶段。另一种推测是联合治疗的抗肿瘤效应更强，恶性细胞数量减少更为显著，虽然大量的免疫效应细胞浸润在瘤床，但并未造成影像学下的肿瘤体积增大。

**表 2:** NSCLC患者的PsP研究

Study	Immunotherapy agents	Study type, No. of patients	Key inclusion criteria	Incidence, n/N (%)
Gettinger et al. (2015)^[14]^	Nivolumab	Dose-escalation cohort extension trial, n=129	1 to 5 prior systemic regimens for advanced NSCLC (platinum- or paclitaxel-based)	6/129 (4.7%)
Borghaei et al. (2015)^[15]^	Nivolumab	Open-label, randomized controlled (Nivolumab vs Docetaxel), n=582	Progressing non-squamous cell NSCLC after first-line platinum-based doublet chemotherapy in stage IIIB or IV	16/292 (5.5%)
Brahmer et al. (2015)^[16]^	Nivolumab	Open-label, randomized controlled (Nivolumab vs Docetaxel), n=272	Progressing squamous NSCLC after first-line platinum-based doublet chemotherapy at stage IIIB or IV	9/135 (6.7%)
Rizvi et al. (2015)^[17]^	Nivolumab	Open label, single arm, n=117	Squamous NSCLC with stage IIIB or IV platinum-based doublet chemotherapy followed by more than one line of systemic therapy	4/117 (3.4%)
Gettinger et al. (2016)^[18]^	Nivolumab	Open label, single arm, n=52	Stage IIIB or IV untreated NSCLC	3/52 (5.8%)
Kazandjian et al. (2017)^[19]^	Anti PD-1 antibody	Retrospective analysis of pooled data from three studies, n=535	Metastatic NSCLC treated with platinum-based doublet chemotherapy	10/535 (1.9%)
Kim et al. (2017)^[20]^	Anti PD-1/PD-L1/CTLA-4 antibody	Retrospective analysis, n=41	Relapsed or advanced NSCLC progressing on platinum-based doublet chemotherapy	2/41 (4.9%)
Gandara et al. (2018)^[21]^	Atezolizumab	Open-label, randomized controlled (Atezolizumab vs Docetaxel), n=850	Stage IIIB or IV NSCLC treated with 1 to 2 lines of chemotherapy (platinum-based doublet chemotherapy)	12/332 (3.6%)
Katz et al. (2018)^[13]^	Anti PD-1 antibody	Retrospective analysis, n=166	Stage IIIB or IV treated NSCLC	3/166 (1.8%)
Tazdait et al. (2018)^[22]^	Anti PD-1/PD-L1 antibody	Retrospective analysis, n=160	Advanced NSCLC progressing after first-line chemotherapy	8/160 (5.0%)
Fujimoto et al. (2019)^[23]^	Nivolumab	Retrospective cohort study, n=542	Previously treated (1-3 line) stage IIIB or IV NSCLC	14/542 (2.6%)
Hendriks et al. (2019)^[24]^	Anti PD-1/PD-L1/CTLA-4 antibody	Prospective cohort study, n=255	Advanced NSCLC brain metastases (containing CNS symptoms)	2/255 (0.8%)

NSCLC: non-small cell lung cancer; PsP: pseudoprogression; PD-1: programmed cell death protein 1; PD-L1: programmed cell death ligand 1; CTLA-4: cytotoxic T lymphocyte associated protein 4; CNS: central nervous system.

双重免疫治疗缺乏PsP发生率的研究报道，但在新辅助双重免疫治疗中，发现了“淋巴结免疫耀斑”现象，类似于纵隔淋巴结PsP。Cascone等^[29]^回顾性分析了NEOSTAR研究中接受新辅助纳武利尤单抗±伊匹木单抗治疗的可手术NSCLC患者（n=44），7例患者淋巴结增大，切除后未发现癌细胞，但出现了新的非干酪样坏死，其中4例（19%, 4/21）接受双重免疫治疗。这种类似淋巴结PsP的情况在化疗联合ICIs新辅助治疗的研究中未有报道。正确识别新辅助免疫治疗后“淋巴结免疫耀斑”现象，可避免一些患者错失手术根治机会，但目前如何鉴别仍是一个难题，对于因淋巴结PD导致不可手术的患者，建议行淋巴结活检证实。

### 2.2 PsP发生时间点

PsP可能发生在免疫治疗启动后的任何阶段，其中多数PsP在免疫治疗启动早期（1-3个月内）出现，71.4%（10/14）患者在继续治疗3个月之内获得了客观反应^[13,23,25]^。PsP发生在第一次CT影像评估时比例更高（67% vs 33%, P=0.04）^[30]^。尽管绝大多数PsP病例报告发生于免疫治疗期间，但停止治疗仍有可能发生^[31,32]^。

### 2.3 PsP表现形式

PsP通常表现为原发灶或转移灶增大，以目标病灶发生多见，也可以表现为非靶病灶进展和出现新病灶^[23,30]^。PsP几乎可以发生在每个解剖位置，主要是与已知病灶存在的部位有关^[33]^，新的病灶需要警惕是否为TPD。Katz等^[13]^研究中3例NSCLC接受ICIs治疗后首次评估出现PsP，其中2例患者表现为原有病灶增大，1例患者出现了新转移灶。Fujimoto等^[23]^回顾性分析了542例接受后线纳武利尤单抗的晚期NSCLC患者，14例患者出现了PsP，其中仅有3例患者出现了新病灶，所有患者均发现原有病灶增大。

其他的PsP表现形式很少见。胸腔积液或心包积液作为PsP表现形式在文献^[34⇓⇓-37]^中被报道，但免疫治疗相关不良事件（immunotherapy-related adverse events, irAEs）也可能导致治疗过程中出现浆膜腔积液，尤其是复发性胸膜腔或心包积液。Kolla^[38]^和Yoshida^[39]^等报道了3例出现PsP的晚期NSCLC患者，这些患者在免疫治疗后出现了胸腔积液暂时增加，原发肿瘤增大，随着免疫治疗继续或者加用糖皮质激素，胸腔积液自发消退，病情得到控制。其中2例患者胸腔积液中都查找到了癌细胞，这导致很难准确鉴别胸腔积液是由PsP还是irAEs导致的。Kolla等^[38]^指出免疫治疗前有胸腔积液的患者更容易发生PsP，尤其是在开始治疗1个月内出现积液增加的患者，随着免疫治疗的继续进行，PsP引起的胸腔积液会逐步缓解，而irAEs导致的胸腔积液则会加重。

PsP通常被描述为放射学检查中病变短暂增大，多不伴有临床症状恶化。Vrankar等^[40]^报道了7例NSCLC接受ICIs治疗后的症状性PsP患者，他们出现的症状包括疼痛、全身炎症反应、上腔静脉综合征、心包填塞、全身恶化、咯血和呼吸功能不全等。这些患者从基线到发生PsP的时间为2-8周，从基线到症状缓解的时间为4-20周。这些症状的出现可能与炎症反应相关，少见的原因可能是原有肿瘤体积增大加剧了占位效应，如上腔静脉综合征、心包填塞等。

### 2.4 PsP的预后

一些研究^[14,22,23,41]^报告了PsP的预后，相较于PD或SD患者，PsP的总生存时间（overall survival, OS）更长。Fujimoto等^[23]^报道，3%（14/542）接受纳武利尤单抗治疗的NSCLC患者出现PsP，预后明显优于PD患者，中位OS分别为未达到 vs 6.4个月（P=0.001），也略长于有客观反应的患者（分别为未达到 vs 20.1个月，P=0.820）。Tazdait等^[22]^描述了160例接受免疫治疗的晚期NSCLC患者中的20例出现了非典型反应，其中8例出现PsP，他们观察到PsP患者的OS比评价为PD患者的OS更长（9.8 vs 6.1个月，P<0.001）。PsP可能是由免疫细胞浸润或延迟性免疫反应导致的，这种情况下患者对继续免疫治疗产生良好的反应，通常生存获益明显，但与典型客观反应患者的预后孰优孰劣尚不清楚。总之，PsP的预后改善与持续免疫治疗获益有关，这意味着准确识别PsP和TPD至关重要，在TPD不能确定，且临床症状和体能状态没有恶化的情况下，继续免疫治疗是值得尝试的方式^[7]^。

## 3 PsP潜在机制的研究进展

PsP发生的机制目前尚不清楚。可能的机制包括免疫细胞浸润、血管变化、肿瘤坏死水肿、免疫反应建立的延迟效应等^[9,42]^。

### 3.1 免疫细胞浸润

ICIs的抗肿瘤作用依赖于机体免疫系统的激活和调动，其中细胞毒性T淋巴细胞相关蛋白4（cytotoxic T lymphocyte-associated antigen-4, CTLA-4）和程序性死亡受体1（programmed cell death protein 1, PD-1）通路是目前研究的两个主要免疫检查点通路。CTLA-4抑制剂在免疫细胞激活早期发挥作用，而PD-1/PD-L1抑制剂并没有提高对肿瘤细胞的直接细胞毒作用，而是促进了免疫细胞的杀伤能力^[43]^。这些药物都需要大量免疫细胞进入肿瘤区域并直接与肿瘤细胞接触，细胞数量的倍增可以使影像上显示的肿瘤病灶体积明显增大。多项研究^[44⇓-46]^ 表明，“一过性、可逆性增大”的病灶活检显示主要为淋巴细胞浸润，例如T细胞和CD103^+^组织残留记忆细胞。因此，推测PsP很可能是由局部组织反应引起的，其病理生理特征为炎症细胞浸润、水肿和血管通透性异常，从而导致CT/MRI上出现新的或更高的对比剂增强，肿瘤体积增大^[10]^。

### 3.2 血管变化

一项关于肺癌脑转移患者的研究^[26]^发现接受ICIs联合放疗比单纯SRS更容易出现短暂而可逆的肿瘤增大（20% vs 5%），可能是SRS导致了血管内皮损伤，通透性增加，免疫细胞更容易浸润，脱髓鞘病变增加，再加上肿瘤坏死水肿，这导致与先前的病变相比强化更明显，范围也较之增大。在此基础上，恶性肿瘤常见的血管系统异常如血管结构紊乱、内皮完整性受损、淋巴引流不良等可能进一步加重肿瘤血管渗漏、瘤周水肿，造成病变体积增大，容易产生PsP^[47,48]^。

### 3.3 肿瘤坏死

放射治疗、化疗、靶向治疗、免疫治疗等会导致肿瘤细胞坏死、凋亡，尤其是在脑肿瘤放化疗患者中，肿瘤坏死可以导致影像学上出现PsP^[49]^。RRx-001（葡萄糖-6-磷酸脱氢酶抑制剂，被认为是一个有效的ICIs）处理的原代异位异种移植物小鼠模型中，重现了PsP的组织病理学特征，其中一些肿瘤由于大面积的中央坏死而体积更大^[50]^。有些肿瘤在接受免疫治疗后通过正电子发射型计算机断层显像（positron emission computed tomography, PET）/CT检查发现体积增大，同时也出现了明显的中央坏死区域，后续随访认定为PsP^[51]^。

### 3.4 延迟效应

免疫治疗延迟效应假说认为，建立有效的抗肿瘤免疫需要一定的时间，这导致ICIs启动后肿瘤仍然继续生长，在免疫治疗发挥功效后肿瘤才开始退缩^[52,53]^。

## 4 PsP病理诊断相关的研究进展

尽管许多研究描述了不同种类肿瘤ICIs治疗后的PsP，但迄今为止，PsP还没有统一的识别标准。活检和组织病理学检查相结合一直是肿瘤诊断的金标准，也用于确认PsP和研究发生机制。

### 4.1 PsP的病理特征

Rocha等^[54]^描述了1例患有PsP的IV期鳞状细胞肺癌患者，接受纳武利尤单抗治疗后表现出不一致的反应（中枢神经系统转移灶PR和肺原发灶SD），但肝脏病变显著进展。肝脏病变的穿刺病理结果显示大面积坏死，没有存活的肿瘤细胞，并且存在淋巴组织细胞浸润。活检显示CD4^+^、CD8^+ ^T细胞增多，且CD4^+^/CD8 ^+^T细胞比例从1.250降至0.875，CD103^+^细胞数量也明显增多，CD68染色显示巨噬细胞比例较高，病理证实为PsP。Curioni等^[55]^报道了1例72岁接受纳武利尤单抗二线治疗的IV期驱动基因阴性的女性肺腺癌患者，有中度腹泻反应，6个周期后PET/CT显示肺原发灶和肾上腺明显缩小，但双肺结节、纵隔、锁骨上淋巴结增大且脱氧葡萄糖（fluorodeoxyglucose, FDG）高摄取，胸膜、多处骨转移灶和肠道均显示出强烈且弥漫的FDG活性。为了区分TPD和PsP，并评估肠道毒性的严重程度，收集了肠道和纵隔淋巴结（4R、7R、11R）的活检组织。结果显示2级ICIs相关性肠炎不伴感染，淋巴结由正常形态的淋巴细胞组成，未发现任何恶性细胞。流式细胞术分析显示30% CD19^+^多克隆B细胞和60% CD3^+ ^T细胞。因此，区域淋巴结的阳性PET/CT信号是由代谢活跃的效应淋巴细胞引起的，而不是真正的肿瘤进展。还有多个经免疫治疗后出现PsP的NSCLC病例通过活检证实，其主要病理表现为肿瘤细胞坏死、反应性炎症变化，淋巴细胞浸润增多（主要为CD8^+^ T细胞），CD4^+^/CD8^+^ T细胞比值较基线下降，且无肿瘤细胞存活^[32,44,56⇓-58]^。这些病理表现被认为是PsP的主要病理学特征。

上述病例中的病理标本主要来源于穿刺活检，但这种取样方式可能具有局限性，大标本或细胞学的PsP病例展示了不一致的病理学表现。Justine等^[59]^报道了1例BRAF V600E突变型转移性黑色素瘤，靶向治疗耐药后出现颅内转移灶进展，在接受帕博利珠单抗治疗后出现影像学进展，手术切除了具有代表性的进展病灶，组织病理学显示出血、反应性星形细胞增多、分散的炎症细胞和大量小胶质细胞混合包围了小簇的肿瘤细胞，其中令人惊讶的是CD8^+^ T细胞很少。Jiang等^[60]^报道了1例二线使用信迪利单抗治疗晚期NSCLC患者，3个周期免疫治疗后，左肺原病灶、肺门和肾上腺转移灶明显缩小，而左肺和右肝出现新发转移，并出现了罕见的牙龈转移。手术切除牙龈病灶证实为转移性低分化肺腺癌，内含丰富的肿瘤细胞。继续免疫治疗后左肺和右肝新发转移灶明显退缩，被认为是PsP。Kim等^[61]^报告了1例二线接受帕博利珠单抗治疗后PsP表现为肠穿孔的晚期NSCLC病例，患者起初并没有发现肠道病变，2个周期免疫治疗后出现了急性腹痛，CT发现十二指肠至近端空肠管壁弥漫性增强增厚、肠穿孔，但原发灶和肾上腺转移灶达到了PR，急诊手术切除穿孔肠段，病理显示肿瘤细胞弥漫性全层浸润和炎性细胞浸润，术后3周复查CT发现小肠壁增厚消退，后续继续免疫治疗。这两个病例对PsP的诊断提出新的挑战，通过病理学判断为“TPD”（即存在大量活性肿瘤细胞）的病灶，可能在临床转归上认定是PsP。这种观点在以新发或复发性浆膜腔积液作为PsP主要表现的病例中得到了支持^[37,38,62,63]^，细胞学证实为恶性胸腔或心包积液，继续免疫治疗之后积液吸收。因此，与免疫治疗前的病理基线对比，反应性炎症变化、免疫细胞浸润增多、活性肿瘤细胞明显减少或消失等病理学改变更有利于诊断为PsP，这可能导致早期活检病理诊断PsP的敏感性下降。

### 4.2 病理诊断的局限性

活检结合病理检查是诊断PsP的金标准，但其临床应用也存在一些局限性。首先，活检属于有创操作，可能会导致穿刺或手术部位严重并发症，例如感染、出血等，有些部位活检风险高而不宜实施。其次，患者对侵入性损伤的恐惧而拒绝活检，依从性差；体能状态不佳也是穿刺的相对禁忌证。第三，存在穿刺失败的可能性，以及活检的标本因肿瘤异质性或样本量小而缺乏代表性，而手术切除的病灶无法进行后续影像学随访，即使继续免疫治疗肿瘤负荷减少也无法确定为PsP还是分离反应所致。此外，进行活检的适当时机也是医生面临的挑战。最后，PsP在病理表现方面也存在着多样性和不确定性，即使组织学或细胞学符合TPD，也应充分结合临床表现、影像学、生物标志物等综合考虑，尤其是小标本取材时。

## 5 PsP影像相关的研究进展

基于传统的CT/MRI检查，对NSCLC免疫治疗后PsP的评估往往需要4-8周的滞后时间进行回顾性诊断，尤其是无法及时准确地与HPD区分，这导致部分患者可能失去挽救性治疗的机会^[64]^。PET/CT、放射组学等新型诊断方式初步显示了识别PsP的研究价值，探索建立新的影像学评估标准。

### 5.1 PET/CT

PET/CT现已用于NSCLC临床分期，使用^18^F-FDG PET/CT来预测免疫治疗疗效在临床上也愈来愈受关注。最常用的代谢反应标准为欧洲癌症研究和治疗组织评估标准（European Organisation for the Research and Treatment of Cancer, EORTC）^[65]^和PET/CT肿瘤疗效评估标准（PET Response Evaluation Criteria in Solid Tumors, PERCIST）1.0版本^[66]^。PERCIST采用针对去脂体重校正的标准摄取值（lean body mass-corrected SUV, SUL），因为技术因素导致的数值变化较小。SULpeak[包含最热体素的1.0 cm直径感兴趣体积（volume of interest, VOI）中的平均SUL值]优于SULmax（代表单个最热体素）。成像中选取5个FDG摄取最高的病灶且不超过2个器官作为目标病灶，SULpeak总和来确定代谢反应。Goldfarb等^[67]^在一项NSCLC队列研究中详细阐述了iPERCIST标准，在PERCIST标准基础上，将首次出现的进展归类为“未经证实的进展性代谢性疾病（unconfirmed progressive metabolic disease, UPMD）”，需要4-8周内再次确认。研究结果显示，相较于无反应者，OS在有反应者中更长（19.9 vs 3.6 个月，P=0.0003），且1年生存率为94% vs 11%。PET/CT对TPD的诊断具有较高的敏感性，尤其是在检测小淋巴结转移和骨转移灶时。在该研究中，46.4%（13/28）患者在第一次评估时归类为UPMD，仅1例患者在后续随访中被认定为PsP。Akhoundova等^[68]^对53例接受ICIs治疗和脑部放疗的NSCLC脑转移患者进行回顾性分析，通过MRI评估对治疗的反应，如果TPD不能确定，则进行^18^F-氟乙基酪氨酸（fluoroethyltyrosine, FET）PET检查，MRI评估显示30例（56.6%）患者至少出现一个转移灶进展，对不能确定的11例患者进行了^18^F-FET PET检查，准确识别出9例（81.8%）患者为PsP。在一项^18^F-FDG PET/CT评估晚期黑色素瘤接受免疫治疗疗效的研究中^[69]^，16.1%（5/31）的患者出现PsP，评估为确定的代谢进展（confirmed progressive metabolic disease, CPMD）患者的肝脾比例（spleen to liver ratios, SLR）平均值明显高于2个免疫治疗周期后PsP的患者（P=0.038）。其中1例PsP患者出现了短暂的、对称的纵隔和肺门淋巴结的结节样代谢异常，这种特殊的代谢改变有助于与TPD的鉴别。这些小样本研究结果表明PET/CT可以用于NSCLC ICIs疗效的评估，对PsP早期诊断敏感性和特异性优于常规影像检查，低SLRmean值、对称性纵隔和肺门淋巴结代谢异常有利于PsP的识别。在NSCLC脑转移瘤患者中，使用特异性高的^18^F-FET示踪剂可以提高鉴别PsP和TPD的准确率。另外，PET/CT评估肿瘤体积增大、肿瘤边缘代谢异常且中央区域大片坏死也可能是PsP的特征表现^[51]^。

考虑到NSCLC亚型之间代谢水平的多样性，例如肺腺癌的葡萄糖代谢水平低于其他NSCLC病理亚型^[70]^，应制定针对NSCLC不同亚型的专门评估标准。机体对抗肿瘤免疫的适应性反应会造成淋巴细胞激活，这可能导致葡萄糖消耗增加，而^18^F-FDG PET/CT可能误判为解剖反应和代谢反应的双重进展^[71]^。因此，开发新指标包括肿瘤代谢、总病变糖酵解等核医学生物标志物在ICIs治疗反应评估方面具有广阔的前景。专门针对PD-L1等免疫检查点的PET示踪剂和特异性探针在预测ICIs反应方面也显示出有希望的结果^[72]^。

### 5.2 放射组学

放射组学从医学图像中提取定量特征，用定量描述符提供肿瘤内异质性信息。放射组学通过对这些特征进行定量和转换分析，可以应用于医学成像分析，包括免疫治疗的疗效评估，从而为精准肿瘤治疗提供一种新颖的非侵入性方法^[73]^。

Barabino等^[74]^使用Delta-Radiomics对33例接受ICIs治疗的NSCLC患者的增强CT进行了分析，确定了27个delta特征能够准确地区分ICIs治疗的放射学反应。此外，还发现9个影像学特征的变化与PsP显著相关。

肿瘤细胞膜上的PD-L1表达是目前NSCLC免疫治疗中最常用的疗效预测生物标志物，表达越高预示疗效越好^[75,76]^。由于瘤内异质性的存在，少数活检样本的免疫组化（immunohistochemistry, IHC）检测结果不能代表整体，而且治疗期间PD-L1的表达可能发生变化^[77,78]^，反复进行侵入性检查获取组织标本难以实现。得益于人工智能领域的进步，放射组学可以助力动态预测PD-L1的表达。Giulia等^[79]^利用CT提取的特征创建了预测NSCLC患者PD-L1表达和肿瘤浸润淋巴细胞（tumor infiltrating lymphocytes, TILs）水平的无创模型。他们发现纹理、效果和边缘与PD-L1表达和TILs直接相关，通过7个CT衍生的放射组学特征可以准确区分肿瘤免疫微环境（tumor immune microenvironment, TIME）炎症型和沙漠型肿瘤，这些特征还与NSCLC患者的预后相关。Khorrami及其同事^[80]^利用135例接受ICIs治疗的NSCLC患者的预处理和随后的CT图像探索了放射组学的潜力，通过与活检样本中的TILs浸润进行比较，证实了放射组学特征与TILs浸润的一致性。TILs数量大幅度增多可能会维持NSCLC PsP的影像学表现^[13,81]^，利用放射组学模型可以早期识别这种情况。

### 5.3 其他影像学进展

动态对比增强的核磁共振成像（dynamic contrast enhanced-MRI, DCE-MRI）是一种可以反映肿瘤血管生成的成像检查方法，通过测量相关的血流动力学参数，它可以定量评估肿瘤组织血液灌注、肿瘤血管生成和微血管通透性。Besson等^[82]^将14例未经治疗且活检证实为NSCLC的患者前瞻性地接受了动态^18^F-FDG胸部PET-MRI（包括DCE），将PET和DCE数据标准化，并拟合到模型中。结果显示NSCLC中的血管和灌注特性在空间上存在差异，动态PET-DCE MRI联合研究可以更准确地定性，识别出高代谢但低灌注/血管化的细胞，这是肿瘤缺氧或高侵袭性的标志，容易与治疗反应导致的肿瘤凋亡和坏死相区别^[83]^。其他的MRI技术，例如超顺磁性氧化铁纳米颗粒T2加权相、弥散加权相、动脉自旋标记和限制性波谱成像等也被证实可以识别PsP^[84⇓⇓⇓-88]^。

总之，关于影像学区分PsP和TPD的研究很多，但都处于探索阶段，需要更高级别的证据来证实这些结论。

## 6 PsP相关生物标志物的研究进展

目前常用的诊断方法早期确认PsP仍具有局限性，需联合多种手段相互补充提高诊断正确率，NSCLC相关的生物标志物如循环肿瘤DNA（circulating tumor DNA, ctDNA）、血清白介素-8（interleukin-8, IL-8）的研究进展为识别PsP提供新的视角。

### 6.1 ctDNA

ctDNA来源于肿瘤患者癌细胞凋亡和/或坏死后的肿瘤DNA，含一定比例的循环无细胞DNA^[89,90]^。PsP患者的ctDNA水平显示出快速而显著地下降，而TPD患者的ctDNA水平则有所上升^[91]^。Ricciuti等^[92]^的研究纳入了接受一线帕博利珠单抗±化疗并接受ctDNA评估的62例晚期NSCLC患者，结果显示放射学评估前ctDNA最高等位基因频率（variant allele frequency, VAF）的快速下降与临床获益显著相关，在基线可检测到ctDNA患者中未发现PsP，仅1例基线ctDNA阴性患者在治疗过程中出现ctDNA阳性（随访第21天），随后的ctDNA检测呈阴性，患者在随访第61天出现肺部病变影像学进展，在随访第81天影像学肺部病变明显改善，符合PsP表现。这说明相对于影像学评估，ctDNA动态监测可以早期预测疗效，可能在更早的时间节点识别PsP。Sanz等^[93]^利用一种肿瘤免疫测定方法评估早期ctDNA动力学，并将其与免疫疗法的早期（I/II期）临床试验结果联系起来。总共涉及37项早期临床研究，采集了来自不同瘤种的81例患者共162份血浆标本，ctDNA在75.3%（122/162）的血浆样本中被检测到，结果显示平均变异等位基因频率（mean variant allele frequency, mVAF）在第2个周期（4周内）较基线时出现下降与更长的PFS（HR=0.43, 95%CI: 0.24-0.77, P<0.01）和OS（HR=0.54, 95%CI: 0.3-0.96, P=0.03）有关，如果mVAF下降>50%，这些差异更明显，而HPD和TPD患者mVAF无明显变化。在该队列中有1例高度微卫星不稳定性的子宫内膜癌患者在接受ICIs治疗后，根据iRECIST标准判定为PD而停止治疗，而4周内ctDNA检测显示所有变异基因的VAF均有显著下降，该患者在这之后没有接受过全身治疗，但截至发稿时仍然存活（OS：44.9个月），这例患者最初的免疫治疗后PD被认定为PsP。另外，有研究^[94]^报道ctDNA预测PsP的灵敏度和特异性分别为90%（95%CI: 68%-99%）和100%（95%CI: 60%-100%）。Goldberg等^[95]^报告了一项通过ctDNA早期评估肺癌免疫治疗反应的研究，ctDNA反应（定义为ctDNA水平较基线至少减少50%）与放射影像反应之间存在高度一致性，至初始ctDNA反应和影像学反应的中位时间分别为24.5和72.5 d，有ctDNA反应者预后明显优于无反应者（中位OS：205.5 vs 69 d；P<0.001）。有2例达到ctDNA清除患者未达到影像学反应，其中1例影像学评估为SD，另1例评估为PD，这2例患者都从免疫治疗获得了长期生存获益。以上研究进展表明，ctDNA水平下降与生存时间延长、肿瘤负荷降低呈正相关，在免疫治疗早期即有很好的关联性，比影像学评估达到客观反应和进展的时间分别提前了约8和10周^[96,97]^，利用影像学检查结合ctDNA动态监测可能会在更早的时间节点准确判断出PsP患者，ctDNA水平较基线明显下降和影像学评估PD同时发生高度提示PsP。

### 6.2 细胞因子

IL-8在招募和激活CXC趋化因子中发挥着重要作用^[98]^。一项研究^[99]^利用多种细胞因子联合来预测肺癌患者对化疗免疫治疗的反应，干扰素诱导蛋白-10（interferon-induced protein-10, IP-10）/IL-8比值增加具有更优的PFS和OS。另一项研究^[100]^旨在评估IL-8水平是否可作为抗PD-1抑制剂黑色素瘤和肺癌患者的预测指标。研究发现，在影像学评估的PsP期间，靶病灶大小增加但同时血清IL-8水平下降。然而，随着患者病情进展，血清IL-8水平稳步上升，这表明IL-8水平可准确反映真实疗效，并可用于帮助区分PsP和TPD。

干扰素-γ（immune interferon γ, IFN-γ）是由CTLs、自然杀伤细胞、自然杀伤/T细胞和γδT细胞分泌的一种抗癌细胞因子，在感染和癌症的免疫应答中起重要作用^[101,102]^。Zhao等^[103]^评估NSCLC（n=40）、胃癌（n=30）患者中血清IFN-γ和IL-5水平与抗PD-1单抗疗效的关系，在客观反应组，IL-5和IFN-γ水平在最佳反应点较治疗前显著降低（P<0.001），与治疗前水平相比，IL-5和IFN-γ水平随着肿瘤进展而显著升高（P<0.001），治疗前较高的IL-5和IFN-γ水平表明NSCLC转移患者的PFS较短（P<0.001）。趋化因子配体2（chemokine C-X-C motif ligand 2, CXCL2）是原癌基因编码的一种促进血管生成的蛋白质，在肿瘤的发生、发展和转移中发挥重要作用。基质金属蛋白酶（matrix metalloproteinase, MMP）2属于MMP基因家族，是一种可降解细胞外基质的锌依赖性酶，能促进肿瘤转移。Matsuo等^[104]^检测了NSCLC患者在PD-1抑制剂治疗前后的外周血中一些可溶性免疫介质的水平，以评估是否与临床预后相关。结果显示，治疗后的血浆MMP2水平上升和CXCL2水平降低均与PFS提高具有显著相关性。此外，3例（4%）获得PsP的患者的CXCL2水平持续下降，低于预处理的基线水平，其中2例的MMP2水平持续上升，高于基线水平。研究者认为外周血CXCL2和MMP2水平监测可能是识别PsP的一种有效手段。

当影像学无法区别PsP和TPD时，血清IL-8、IL-5、IFN-γ和CXCL2水平下降有助于诊断PsP，MMP2水平持续升高也可能预示着PsP。

### 6.3 调节性T（regulatory T, Treg）细胞

Treg细胞可抑制恶性疾病中针对癌细胞的免疫反应，从而起到免疫抑制的作用。一项研究^[105]^利用流式细胞学检测NSCLC患者接受ICIs治疗前后的Treg细胞水平变化，在ICIs治疗后PsP发生率为13.5%，HPD发生率为8.1%，治疗开始后7 d外周血中CD4^+^CD25^+^CD127lo FoxP3^+^ Treg细胞在PsP患者中与基线相比明显减少（P=0.038），而在HPD患者中明显上升（P=0.024）。在应答组中，CD4^+^CD25^+^CD127loFoxP3^+^ Treg细胞和PD-1^+^CD4^+^CD25^+^CD127loFoxP3^+^ Treg细胞在治疗开始7 d后相较基线水平显著下降（分别为P=0.034和P<0.001）。这提示循环Treg细胞是一种很有前途的潜在动态生物标志物，可预测NSCLC患者免疫治疗后的疗效，并区分非典型反应，与基线水平相比，在PsP患者中Treg细胞水平显著下降，而发生HPD时则明显上升。

## 7 结论

随着ICIs在NSCLC患者中使用越来越普遍，PsP逐步为临床医师所认识。但关注PsP的研究并不多，在定义、发生机制、鉴别诊断、预测指标等方面仍存在许多亟待解决的问题。PsP发生机制并不明确，目前也缺乏广泛认可的定义，通常认为是免疫治疗启动后肿瘤最初尺寸增大或出现新病灶，随后肿瘤负荷下降或稳定的现象。这种肿瘤负荷的变化主要依赖于影像学连续评估，准确诊断在时效上具有延迟性。病理学确定的新发转移灶或新发恶性浆膜腔积液不能直接排除PsP，继续免疫治疗后疗效评价可能会改善。PET/CT、放射组学、ctDNA动态监测、细胞因子变化等有助于早期识别PsP，但均处于探索阶段，缺乏大规模的前瞻性研究证实。PsP患者继续使用免疫治疗可获得良好的预后，但在临床症状恶化或合并irAEs的患者中，继续使用ICIs需要慎重选择，尤其是怀疑HPD的患者，早期准确诊断极为重要。因此，我们需要在定义上达到统一，开展更多的相关临床研究，在发生机制、诊断方法等方面进行深入探讨。
